# Contributions of Volunteer Community Mobilizers to Polio Eradication in Nigeria: The Experiences of Non-governmental and Civil Society Organizations

**DOI:** 10.4269/ajtmh.19-0068

**Published:** 2019-10

**Authors:** Janefrancis Ijeoma Duru, Samuel Usman, Opeyemi Adeosun, Katherine V. Stamidis, Lydia Bologna

**Affiliations:** 1 Gender Care Initiative, Lagos, Nigeria;; 2 CORE Group Partners Project, Abuja, Nigeria;; 3 CORE Group Polio Project, Washington, District of Columbia

## Abstract

The Northern states were the epicenter of the wild poliovirus outbreak in Nigeria in 2016. To raise immunization coverage, particularly of polio, the Polio Eradication Initiative (PEI) in Nigeria introduced the use of nongovernmental organizations and volunteer community mobilizers (VCMs) through the CORE Group Polio Project (CGPP). The CGPP has been contributing to Nigeria’s polio eradication efforts since 2013. This article explores the contributions of the 2,130 VCMs deployed in 31 participating local government areas in the five implementing CGPP states from 2014 to 2017 to increase awareness, understanding, and acceptance of polio immunization. Data for the study were collected from primary and secondary sources using five collection methods: a survey of VCM supervisors, focus group discussions with VCMs and their supervisors, key in-depth interviews with community stakeholders, case studies of specific best practices of VCMs, and a review of documents and records. A review of the data shows that the VCMs received comprehensive training on the importance of the PEI, routine immunization, Acute Flaccid Paralysis (AFP) surveillance, social mobilization and community engagement, use of behavior change communication tools, and interpersonal communication skills. According to the data collected, the VCMs used the following innovative strategies to ensure high vaccination coverage: house-to-house mobilization, community dialogues, compound meetings, community health camps, and tracking of non-compliant families, missed children, and dropouts. The involvement of VCMs in Nigeria’s PEI efforts has been a pivotal contribution to reductions in the number of households rejecting polio immunization, the proportion of families with missed children, the proportion of families that were non-compliant, and the number of polio cases.

## INTRODUCTION

Vaccination has been one of the greatest public health achievements from the early twentieth century to the present. Vaccines have prevented millions of deaths and cases of serious morbidity, and they are among the most cost-effective public health measures available.^1^ According to immunization rates provided by the World Health Organization (WHO) in 2018, immunizations prevent at present more than 1.5 million child deaths each year across the globe.^2^ Despite these immense benefits, vaccination programs in Nigeria, especially those for childhood vaccination, are besieged by multiple challenges, including underutilization of immunization services, lack of sustainable financing, and misleading information on vaccines and their effects.^3^ In Nigeria, vaccine-preventable diseases account for approximately 22% of child deaths in the country.^4^ Although vaccination rates have increased in the past decade, children in the northwest are nearly three times less likely to receive all basic vaccinations than children in the southeast (20% versus 57%).^5^ These low immunization rates have been attributed to vaccine hesitancy (caused by lack of trust in the vaccine or the provider, misunderstanding of the need for vaccination or of the effects of vaccination, religious beliefs, and rumors), poor access to health facilities, fear of violence, lack of knowledge, illiteracy, and other social and political factors.^6^

This article reviews the Polio Eradication Initiative (PEI) in Nigeria and the use of civil society organizations (CSOs) and nongovernmental organizations (NGOs) in the PEI, and highlights the work of volunteer community mobilizers (VCMs) that have been deployed by the CORE Group Polio Project (CGPP). The CGPP is described in another article in this series,^7^ and Nigeria’s community engagement is discussed in another article.^8^ This article addresses the role and relevance of VCMs working with NGOs and CSOs, their performance, and their contributions to the PEI. Specifically, the article seeks to answer the following questions:

1. What informed the use of CGPP VCMs for polio eradication in Nigeria?
2. What have been the innovative strategies used by CGPP VCMs for polio eradication in Nigeria?
3. What have been the contributions of CGPP VCMs to the eradication of polio in Nigeria?

## OVERVIEW OF THE PEI IN NIGERIA

The 41st World Health Assembly in 1988 adopted a resolution for the global eradication of polio and concomitantly launched the Global PEI (GPEI). At the GPEI’s start in 1988, Nigeria’s immunization programs were affected by barriers typically found in low- and middle-income countries, including inadequate funding, low coverage rates, poor supervision, broken cold chains, and a lack of community mobilization and engagement.^3^ To achieve the eradication target, Nigeria joined other affected countries to develop and deploy specific polio eradication strategies including Acute Flaccid Paralysis (AFP) surveillance, national immunization days (NIDs), and supplementary immunization activities (SIAs).

From the onset, Northern Nigeria presented an extreme challenge. The transmission of polio in Northern Nigeria was due to complex health, economic and social issues such as poor demand for and access to health services, low immunization coverage, few available skilled health workers, extreme poverty, low literacy, and community resistance to immunization and government services.^9^ Other factors such as the safety of the vaccine, religious factors, and community distrust of government health systems played a major role in increasing transmission.^10–14^ This led to a reemergence of polio in Nigeria, especially in the Northern states.^15,16^ Even in areas where polio immunization was not controversial, failure to engage parents and discuss why a fully vaccinated child may develop polio disease, for instance, reinforced and increased parents’ negative perceptions of the polio program. There was a need to strengthen understanding of the importance of vaccination and to rebuild community trust and support for the program.^17^

**Figure 1.**
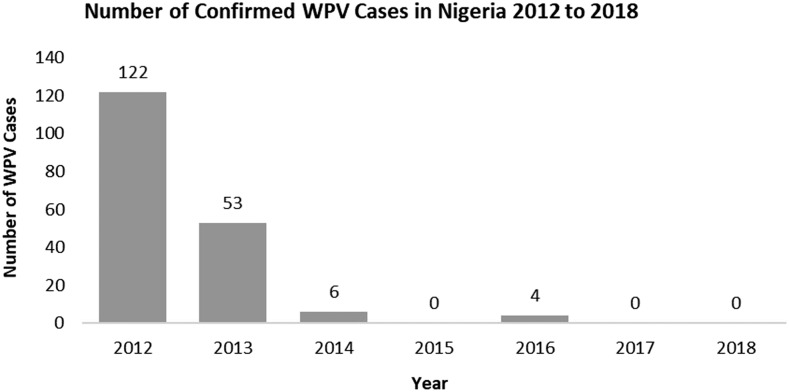
Number of confirmed wild poliovirus cases in Nigeria, 2012–2018. Source: Nigeria’s National Polio Emergency Operations Center 2018.

In 2009, 388 new wild poliovirus (WPV) cases were reported in Nigeria, according to AFP polio surveillance data from the National Primary Health Care Development Agency.^18^ There was a gradual decline in the cases such that by 2013, only 53 cases were recorded (Figure 1). Also, in 2009, a total of 154 circulating vaccine-derived poliovirus (cVDPV) were reported across the country. Mimicking the decline of WPV cases in 2013, only one case of cVDPV was reported that year. Even with the decline in the cases of new WPV and cVDPV cases reported in 2013, Nigeria accounted for more than 50% of the cases reported globally in 2012.^19^

In late 2013, the CGPP began work in Nigeria. The CGPP is a multicountry, multi-partner initiative that provides financial support and on-the-ground technical guidance and support to strengthen country efforts toward polio eradication through NGOs and civil society. With a global secretariat based in the United States, the CGPP provides technical assistance and financial management to project countries. To maximize and harmonize resources and coordinate collaboration among partners.^7^ In Nigeria, the CGPP is referred to as the CORE Group Partners Project (hereafter also referred to as CGPP) to de-emphasize the use of the word “polio” in sensitive communities.

The CGPP joined the federal government and United Nations Children’s Fund (UNICEF) to use VCMs in 2014. This new initiative introduced unique strategies and operated through CSOs and NGOs. The CGPP engaged VCMs to increase awareness, understanding, and acceptance of polio immunization. Three years later, the proportion of missed children and the proportion of noncompliant families, along with the number of polio cases, all declined in most local government areas (LGAs) in the polio-prevalent Northern states.^20^ The last two reported cases of WPV were in July 2014 and no cases in 2015. In mid-2016, the WHO declared Nigeria a polio non-endemic country.

However, in August 2016, after more than 2 years without detection of WPV in Nigeria, the government discovered two cases of WPV in the Monguno LGA of Borno State, a previously inaccessible area. In response to the outbreak, multiple vaccination campaigns were held to rapidly raise population immunity and prevent further spread of the virus. The campaigns were held not only in Nigeria but also in neighboring countries, particularly in the Lake Chad subregion, including northern Cameroon, parts of Central African Republic, Chad, and southern Niger. The CGPP played an active role in the planning of these campaigns, and the CGPP has since continued with a strong presence in high-risk areas. The CGPP used multipronged approaches to reach children in the most challenging circumstances. Vaccination was conducted during periods of improved security and at markets, at cross-border points, and during outreach to nomadic populations.

There were four WPV1 cases in 2016, and, as of July 2019, no further cases of WPV had been reported. However, suboptimal vaccination coverage in high-risk locations has resulted in multiple cVDPV type 2 outbreaks. The continued vigilance and commitment to maintaining high vaccination rates are necessary to ensure that immunity levels among susceptible children younger than 5 years remain high to prevent transmission of poliovirus.

### Use of civil society and NGOs in the PEI.

Civil society organizations and NGOs are a very diverse collection of local, national, and international entities, including professional associations and academic institutions. With immunizations, the broad role of CSOs and NGOs involves provision of services; demand creation; disease surveillance; and advocacy for equitable access, affordability, funding, accountability, and improved quality of services. The work of CSOs and NGOs at the community, district, national, regional, and global levels to increase access to immunization is critical to reaching every vulnerable child, saving children’s lives, and improving people’s health.

The CGPP has promoted and championed the inclusion and contributions of civil society to global polio eradication using the CGPP Secretariat Model, which coordinates a group of NGOs under the umbrella of a national secretariat office with links to other partners and the government within their project countries. The Secretariat Model is described in further detail in another article is this series.

**Figure 2.**
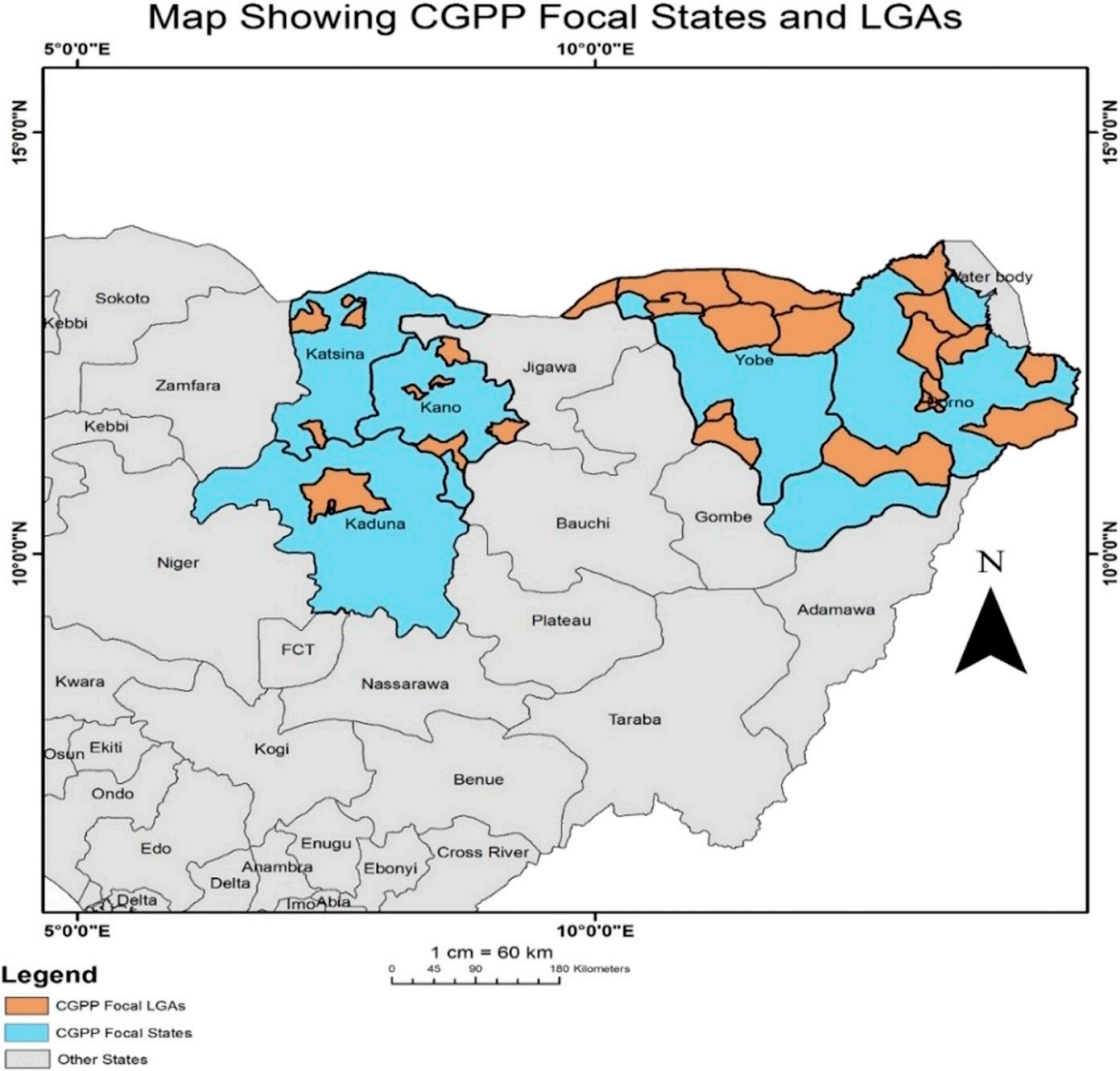
Geographic areas in Northern Nigeria where the CGPP is working. Source: CGPP Nigeria Annual Report 2018.^21^

The Nigeria/CGPP began its preliminary polio eradication activities in 2013 in the high-risk priority Northern states of Kano, Kaduna, Katsina, Borno, and Yobe (Figure 2). These insecure areas are home to the Boko Haram terrorist insurgency group, and these states have long struggled to increase immunization rates. During the formative phase of this program in late 2013, the CGPP developed polio eradication activities in Nigeria through the National Polio Emergency Operations Center (EOC) (and more specifically through the National Polio Eradication Emergency Plan) in high-risk areas by integrating with ongoing child health services. CGPP/Nigeria joined other implementing partners under the general operational auspices of the national and state EOCs. CGPP/Nigeria launched full implementation of activities in January 2014.

Polio vaccination is provided through a variety of approaches, including Immunization Plus Days (IPDs), Directly Observed Polio Vaccination campaigns (held a couple of days before house-to-house vaccination), Immunization Between Rounds (held after a campaign for underperforming settlements), hit and run campaigns, Reaching Every Settlement (RES), Reaching Inaccessible Children (RIC), outreach health camps held for immunization of children located in internally displaced camps, the use of permanent vaccination teams,† and transit point vaccination for nomadic populations.^22,23^ Hit and run campaigns are based on the principle of ensuring maximal immunization of children younger than 5 years living in security-compromised areas. These campaign rely on teams that rush into insecure communities during brief periods of relative calm to “hit” (i.e., immunize) children and then “run” (i.e., rush out before security worsens again). Similarly, RES and RIC strategies are used in security-challenged areas and involve the use of government military and rebel groups to deliver immunizations to children. These strategies have been used in CGPP focal settlements in Borno and Yobe to reach children who could not be reached by more traditional methods. Reaching every settlement was implemented in partially accessible settlements with good progress. Reaching inaccessible children was used in fully inaccessible areas with equal success. The CGPP supported these strategies through planning and implementation in Borno State. An earlier sentence states CGPP/Nigeria used these strategies both in Borno and Yobe.^21^

**Figure 3.**
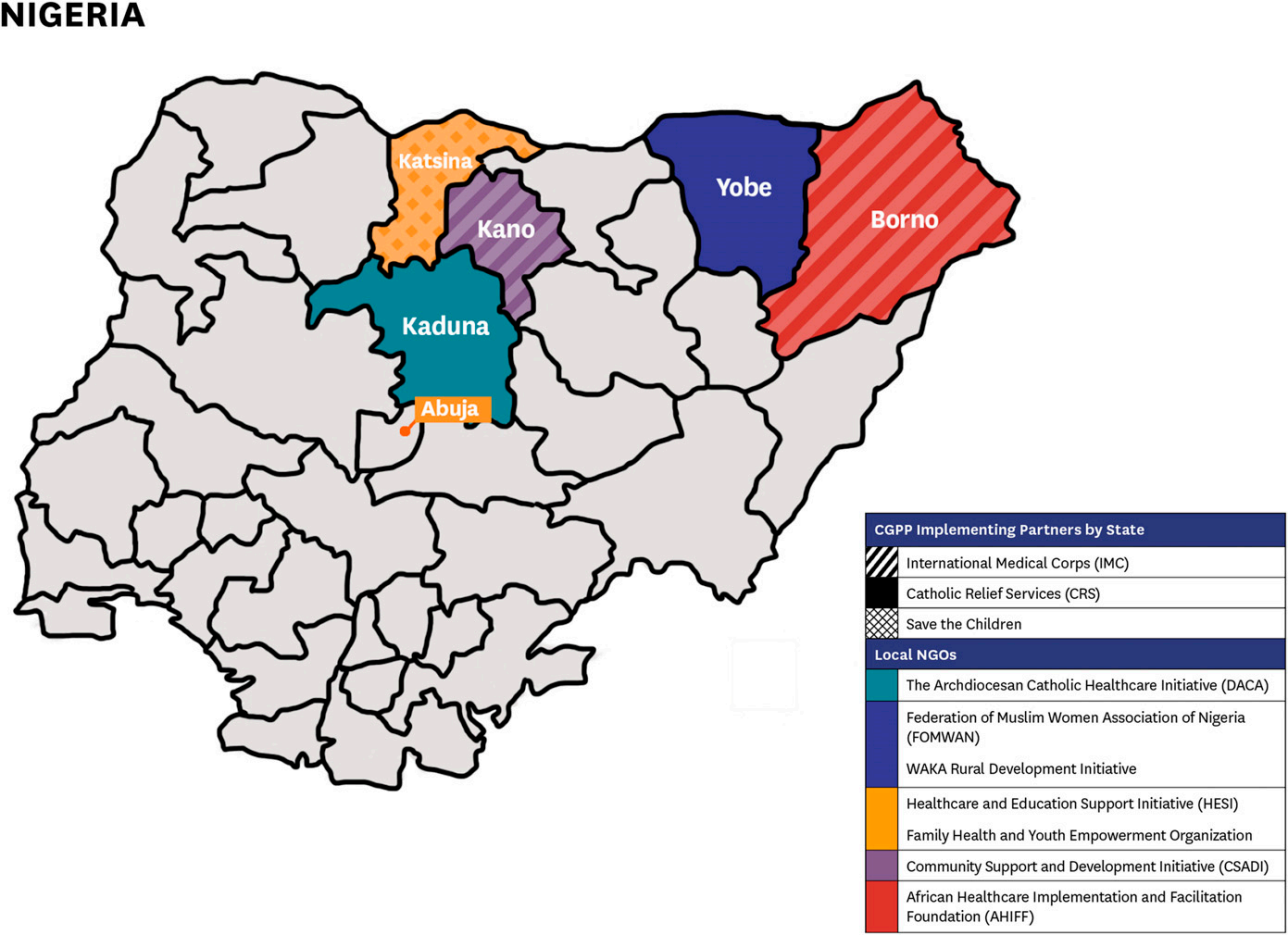
The three international non-governmental organizations (NGOs) and seven local NGOs in each of the five Northern states.

In Nigeria, the CGPP partners with three international NGOs: Catholic Relief Services, International Medical Corps, and Save the Children. In turn, these international NGOs partner with a total of seven local community-based NGOs by providing supervision and direction. The seven local groups are the Archdiocesan Catholic Healthcare Initiative in Kaduna State; the Federation of Muslim Women Association of Nigeria and Waka Rural Development Initiative in Yobe State; Family Health and Youth Empowerment Organization and the Healthcare and Education Support Initiative in Katsina; Community Support and Development Initiative in Kano State; and African Healthcare Implementation and Facilitation Foundation in Borno State (Figure 3).^22^

### Documenting the contributions of Volunteer Community Mobilizers in Nigeria’s PEI.

**Figure 4.**
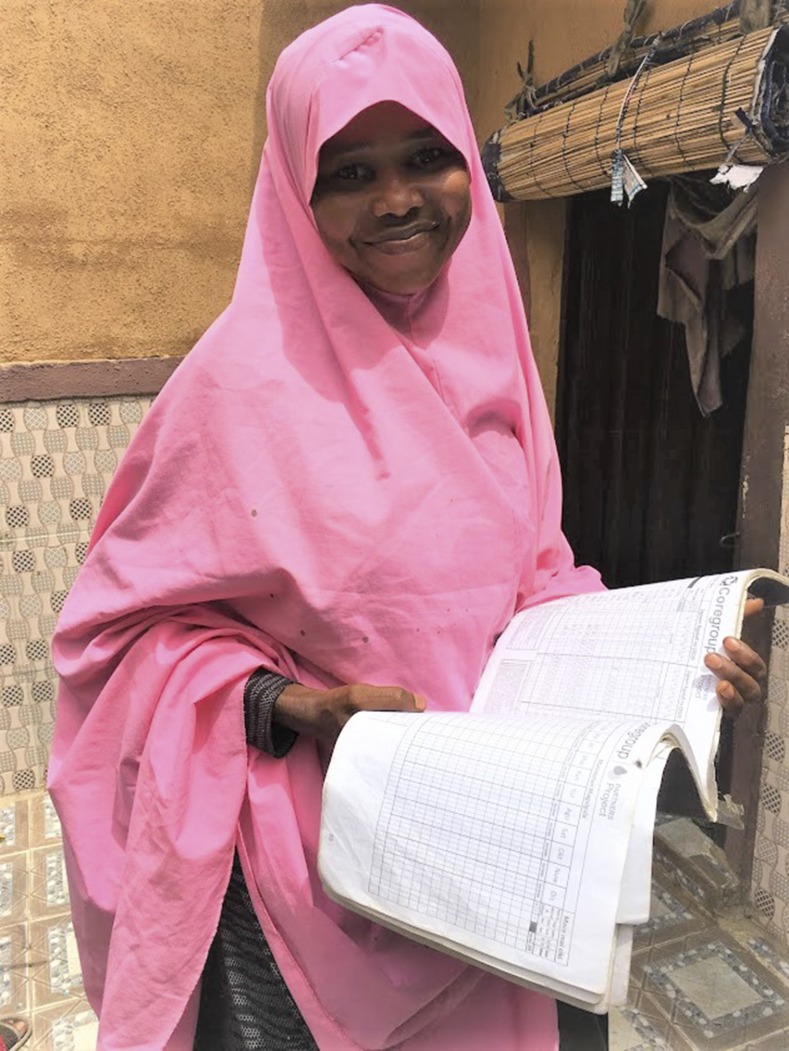
VCM Maryam Bala, who works in Kano State’s Nasarawa LGA, reviews her community register to check the immunization status of the children under five years old living in the compound. Ms. Bala, 27, has been a CGPP VCM for four years. Photo credit: Lydia Bologna, CGPP.

The use of VCMs by the CGPP in Nigeria started in 2014 (Figure 4). This was in direct response to the widespread rejection of oral polio vaccine, unprecedented non-compliance, child absenteeism at immunization points and during campaigns, high numbers of missed children, and the high immunization dropout rate. The primary role of VCMs was to mobilize caregivers in all households for “Massive Health Promotion” and increase the awareness of caregivers about vaccine-preventable diseases and the availability of free immunizations. The VCMs were tasked with tracking and resolving routine immunization dropouts, non-compliant families, and defaulters, as well as strengthening surveillance for AFP. In addition, they were charged with promoting key household practices related to nutrition and water, sanitation, and hygiene (WASH). More recently, greater attention has been focused on the contributions of VCMs who work to improve health outcomes among their peers living in exceptionally difficult areas of Northern Nigeria. The scope of their performance and the extent of their impact have been previously reported.^24,25^ Our article adds to the literature by documenting the specific contributions and key strategies used by the CGPP VCMs.

## METHODS

### Study design.

A mixed-methods design was used for this study. Quantitative and qualitative data were used from both primary and secondary sources. Triangulation of data allowed for a more comprehensive description and analysis of the practices that VCMs used to contribute to PEI in Nigeria.

### Data collection and instruments.

Data for the study were collected from a total of four data sources. Primary data were collected through a survey of program supervisors, focus group discussions (FGDs), and key in-depth interview (KIIs) between August 27, 2017 and September 3, 2017 (Table 1). Secondary data were obtained from a review of documents and records.

**Table 1:** Summary of collection of primary data

Data collection instrument	Number of participants	Participant description
Semi-structured questionnaire	20	Five state team leads and 15 volunteer ward supervisors
Focus group discussion (FGD)	51	Three FGDs held with 36 VCMs randomly selected from three wards in two local government areas in Kaduna State (12 VCMs per ward).
		Three FGDs held with 15 VWSs from Kaduna, Yobe, and Katsina (five VWSs per state)
Key in-depth interview	12	Twelve community stakeholders (district heads, district secretaries, and women leaders)

VCM = volunteer community mobilizer; VWS = volunteer ward supervisor.

Qualitative data were collected through FGDs with VCMs and their supervisors known as Volunteer Ward Supervisors (VWSs) and through KIIs with community stakeholders. Quantitative data were collected through survey questionnaires with program supervisors—VWSs and State Team Leads (STLs). The document review consisted of examining program reports, policy papers, and other related literature. Case studies allowed for an in-depth analysis of the specific best practices used by the VCMs.

#### Primary sources.

The survey consisted of a self-administered, semi-structured, standardized questionnaire completed by program supervisors (five STLs and 15 VWSs). The survey questionnaires consisted of dichotomous, multiple-choice, and open-ended questions. A combination of these techniques was intended to maintain the interest of the program supervisors. Both questionnaires were pretested. The Supplemental Appendix contains a copy of the two questionnaires used for the study.

In all, six focus groups were held with a total of 51 participants (Table 1). Three FGDs were held with randomly selected VCMs from three different wards in Kaduna State. Three additional FGDs were held with VWSs from Kaduna, Yobe, and Katsina States. The lead author led the discussions. Two research assistants worked with the lead author: one orally translated the conversation (questions, answers, and explanations), whereas the other took notes of the discussion.

A semi-structured questionnaire was administered to the five STLs and the VWSs from Kaduna, Yobe, and Katsina. Key in-depth interviews were conducted with community stakeholders to assess their opinion on the contributions of VCMs to their roles and contributions polio eradication. Stakeholders included district heads, district scribes (secretaries), and women leaders. The interviews were semi-structured to allow for flexibility. The questions were open-ended, encouraging respondents to share information freely. The interviews were conducted in the Hausa language, captured by digital recordings, and then translated into English written notes.

#### Secondary sources.

Secondary sources of data used in our analysis included written documents and records on polio, routine immunization, and the use of VCMs. The review included government documents, institutional publications, statistical reports, journal articles, and annual program reports. Information collected from these sources was used to compare and enrich the data collected from primary data sources.

#### Study sites.

This study was conducted in the five implementing states of Kaduna, Kano, Katsina, Yobe, and Borno. The main study sites were three wards in two LGAs in Kaduna State where we administered questionnaires and conducted FGDs with VWSs, VCMs, ward scribes (secretaries), and women leaders.

#### Study population.

The populations for the study were the VCMs, their supervisors (VWSs), the LGA coordinators (LGACs), and the STLs.

### Sampling methods.

A purposive sampling method was used to select respondents for the KIIs. We used a random sampling method to select VCMs for the FGDs. A semi-structured questionnaire was administered to the five STLs and the 15 VWSs from the three implementing states. A total of 36 VCMs were randomly selected for the FGDs. The purpose of the research was explained to the VCMs before conducting the FGDs. The research team also discussed the study objectives with the VCMs who worked in the primary health-care facilities of the three wards selected in both LGAs. These were the health facilities under which the VCMs worked. Key in-depth interviews were also held with community stakeholders (village heads, district scribes [secretaries], and women leaders) in the three selected wards.

### Data analysis.

Data were compiled through tabulation of the survey questionnaires. Information was also extracted from open-ended questions. The data obtained from the FGDs were analyzed using an inductive approach: a content analysis was performed and a simple descriptive narrative was written. We also triangulated qualitative evidence (including extensive field notes) with published program reports, policy papers, and published literature.

### Ethical considerations.

The study observed the key principles of research ethics. Voluntary informed consent was obtained from participants before data collection. Participants’ autonomy to respond was respected. Privacy and confidentiality of the information were maintained by concealing identifying information after the data had been collected.

## RESULTS

According to the questionnaire administered to the STLs, a total of 2,250 VCMs were selected from the communities in the 32 participating LGAs in the five implementing states (Table 2). The STLs reported that the implementing LGAs were selected after meeting with the State Polio Emergency Operation Centre and Primary Health Care Development Agency using the following criteria: high-risk LGA, high number of noncompliant households and unimmunized children, low IPDs participation (for polio), low routine immunization coverage, LGA with hard-to-reach settlements, and low AFP surveillance reporting.

**Table 2:** Implementing states, number of local government areas (LGAs), and number of CGPP VCMs participating in the data collection

State	Number of implementing LGAs	Implementing LGAs	Number of volunteer community mobilizers
Kaduna	2	Igabi and Kaduna North LGAs	100
Kano	6	Dambatta, Nasarawa, Takai, Tudun Wada, Rimin Gado, and Ungogo	320
Katsina	4	Batsari, Funtua, Katsina, and Rimi	270
Yobe	10	Bursari, Geidam, Karasuwa, Fika, Fune, Machina, Potiskum, Nguru, Yunusari, and Yusufari	780
Borno	10	Abadam, Damboa, Guzamala, Kala–Balge, Konduga, Jere, MMC, Monguno, Ngala, and Nganzai	660
5	32	–	2,250

Source: CGPP Final Evaluation Report 2017.^21^

All of the VCMs are females residing in the communities where they work. They are well respected, have knowledge of the community’s culture, can speak the community’s local language fluently, and to some extent can read and write and have knowledge of polio immunization. It is mandatory for VCMs to wear CGPP-branded pink hijabs to respect the norms, culture, and values of their community. Moreover, the hijabs function as both the dress code for VCMs and a form of community identification and recognition. A VWS from Yobe State said:

The VCMs are known and referred to as pink women. The hijab also provides them with some sort of security and indicates that VCMs are friends of the family and community.

A VCM is typically assigned to one community, although in some cases two VCMs are assigned to a community based on its unique qualities or population size. Each VCM is assigned between 150 and 300 households in her community. To garner support for the program, the VCMs and VWSs pay advocacy visits to the community leaders, religious leaders, opinion leaders, women leaders, and other stakeholders. Using project champions, they also mobilize the community and organize forums to create awareness and gain community acceptance and approval.

According to the STLs and VWSs who were interviewed, VCMs receive comprehensive, quality training. The CGPP organized several trainings for the VCMs in their communities on the PEI, routine immunization, AFP surveillance, social mobilization and community engagement, use of behavior change communication tools, interpersonal communication, SIAs and IPDs, data collection, and on other health issues beyond polio. After their training, the VCMs were equipped with essential logistical and promotional materials such as register books, flip charts, and other behavioral change communication (BCC) tools for delivering health messages. One VWS described the usefulness of the social mobilization materials given to the VCMs during a FGD:

They use the flipcharts, danglers, and so forth to engage caregivers with behavior-changing dialogue. The charts contain messages on polio vaccination as well as other key household practices such as treatment of diarrhea, prevention of malaria, breastfeeding, and hand washing, thereby creating a trusting relationship with the caregivers.

On regular days, VCMs work individually with families in their assigned communities, documenting immunization status, recording pregnancies and births, and providing information on vaccination and other health topics. The rapport they develop with their families is paramount. VCMs work for a minimum of three 8-hour days (or 24 hours per week), although many work longer hours during periods of heightened activity. One of the VWSs reported that:

During immunization campaigns or microplanning, VCMs work throughout the week to ensure that immunization messages and vaccines reach every child.

Before the beginning of SIAs, VCMs participate in microplanning to provide useful information on the community and data on defaulters. During IPDs or during SIAs, VCMS work in four-person teams: a vaccinator, a recorder, a community leader, and the VCM. A supervisor oversees each team. VCMs note refusals and supervisors report on these at LGA meetings during the SIAs. The VCMs’ job is not full time; most of them have other means of livelihood. However, they receive monetary stipends of about $21 per month as well as nonmonetary incentives such as training opportunities, umbrellas, bags, and a uniform.

### Reaching communities through effective strategies used by VCMs.

According to reports from the VWSs and VCMs at the time of the FGDs and corroborated from questionnaires completed by both STLs and VWSs, the innovative strategies used by the VCMs include house-to-house mobilization, community dialog, compound meetings, community health camps (a week-long health campaign where health services including immunization are offered to the community for free), keeping community registers, tracking cases of non-compliance, locating missed children and dropouts, accompanying mothers to a health facility for immunizations and other health services, using BCC tools, and providing health education (Table 3).

**Table 3:** Effective strategies used by VCMs

Strategies	Explanation
House-to-house mobilization	This consists of in-person interactive visits conducted by VCMs to create awareness of and to increase demand for immunization services and other key household practices in their assigned communities.
Naming ceremony	The traditional 7th day naming ceremony, also known as *Suna* Immunization, is an activity during which a VCM immunizes the newborn child and other children younger than 5 years. This traditional ceremony is organized by the family that invites relatives, friends, and well-wishers. The VCM, with the support of her supervisor (the VWS), collects oral polio vaccine from the health facility and joins the family in the celebration. During the ceremony, the newborn receives the birth dose of OPV as do the other young children who are in attendance.
Meeting of compound members	This is an interactive activity between the VCM, a higher level CGPP program staff member, and members of immunization-resistant families within a household or compound. This meeting targets mainly women but could include men if circumstances permit. During the meeting, the VCM explains the purpose and importance of the Polio Eradication Initiative and the implications of not immunizing.
	Members of the compound can voice their concerns and fears, which most times emanate from myths and misconceptions about immunization. These concerns are passionately addressed by the VCM and the accompanying CGPP staff member. In most cases, the meeting results in the acceptance of immunization services by the vaccine-resistant families.
Community dialogue	This is a meeting between CGPP program staff members and important members of the community to address the challenges to obtaining immunizations and other health-care services and to performing key healthful household practices. A community dialogue is typically organized when there are many non-compliant or resistant families after supplemental immunization activities despite relentless efforts by the VCMs during the house-to-house mobilization to convince them to accept immunization. The meeting is convened in the affected community by the local government area coordinator or a higher level program staff member, with the VCM in attendance. If the challenges are resolved, the immunization teams revisit the households to immunize the children. In some cases, the CGPP team will arrive for follow-up with the health officer who then vaccinates all children younger than five years with OPV and immunizes children younger than 1 year with the other routine vaccines.
Community registers	These registers are maintained by the VCMs. The registers are used to document the vaccination history of all children and track pregnant women and newborns, and are used by mobilizers to encourage parents to seek routine immunization for their children at prescribed intervals. Data from these registers are sent from the local level to the state and secretariat level on a monthly basis. Community registers are, perhaps, the most successful documentation strategy used by Nigeria/CGPP.

CGPP = CORE Group Polio Project; VCM = volunteer community mobilizer.

VCMs deliver their messages verbally in coordination with a variety of BCC tools such as pictorial flip-books, stickers, streamers, banners, posters, danglers, wrist bands, fliers, and T-shirts. The BCC tools address the importance of vaccination and provide general knowledge on the immunization program, the safety of oral polio vaccine (OPV), the immunization target population, the number of doses needed for each vaccine, and the route of administration; and, finally, they provide useful information about personal hygiene and nutrition.

The VCMs do their routine work in the households and the village squares. During IPDs and State Immunization Polio Days, they visit and work at schools, churches, mosques, motor parks (bus terminals), and markets. In the words of one of the VWSs:

The VCMs also use town announcers, a papalolo [a local clown that is used to attract people’s attention at events], and short dramas to get people’s attention and to disseminate their messages during this period.

The VCMs also work with the health facilities to refer or escort missed children to the health center for immunization and to connect non-compliant parents with health facility personnel. The traditional birth attendants, traditional healers, bonesetters, traditional barbers, and chemists work with the VCMs as community informants. These informants are trained to identify major childhood diseases (such as measles, malaria, and malnutrition) and conduct surveillance for AFP. The VCMs hold monthly meetings with the community informants to provide refresher training, discuss challenges, and reinforce positive achievements.  The VCMs also work closely with community informants and their VWSs to increase AFP case detection in their communities. A report from Katsina STL supported this:

A VCM working with a VWS in Batsari LGA reported six cases of AFP within two months after several trainings/interactive sessions on signs and symptoms of AFP and other health issues with the community informants.

CGPP VCMs have made a notable contribution to polio surveillance in project areas. During fiscal year 2018, CGPP VCMs, along with CGPP-trained Community Informants, identified 44% (160/364) of the AFP cases reported from project catchment areas. In addition, the non-polio AFP detection rate rose from 13.6 to 19.6 cases among children aged 0 to < 15 years of age per 100,000 populations between 2014 and 2017, much higher than the expected rate of 2.0.^21^

The VCMs work in close alliance with highly respected community leaders who serve as the bridge between the VCMs and their communities. The community leaders not only assist in selecting respected and influential women to serve as VCMs but also help to resolve cases of immunization defaulters or rejections and to mobilize their communities for immunization activities. The community leaders participate in planning meetings and community dialogs. A community leader from Ungwam Shanu explained this during an interview:

Since the program started working with VCMs who are known and respected women from our communities, there has been a great improvement in the immunization program: the number of cases of rejections and missed children in the communities have declined and, in most communities, do not recur. This is because we now understand the importance of their work, support their activities, and ensure compliance by our people.

**Figure 5.**
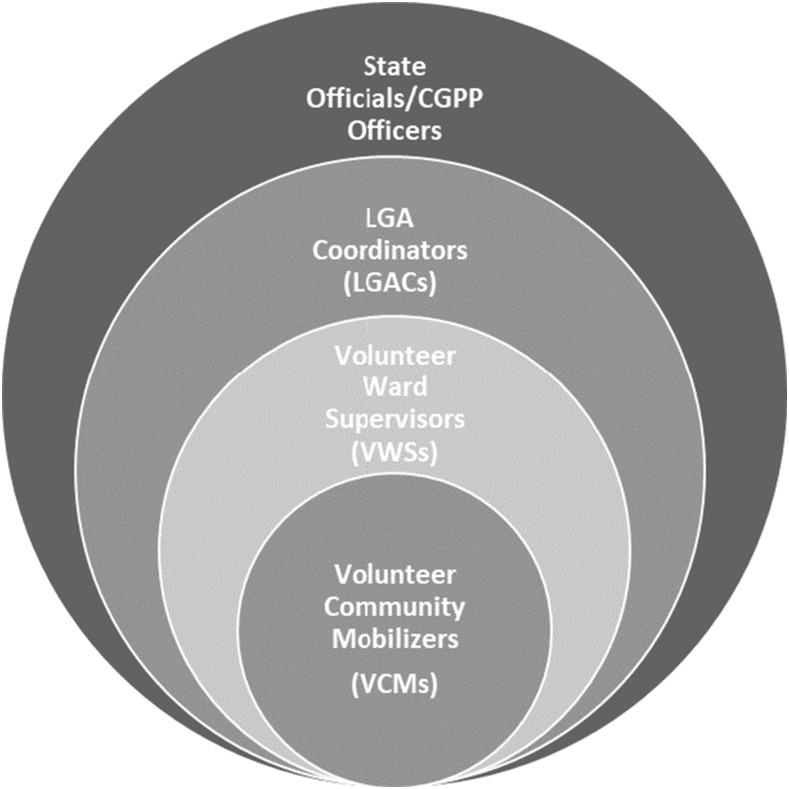
Multilevel supervision of the VCM network.

Multilevel supervision is a key strength of the CGPP VCM network and is a contributing factor in the program’s success (Figure 5). The first level of supervision is provided by the ward focal person, also known as a VWS. The VWS regularly monitors and supervises the activities of the VCMs to ensure coverage of their settlements. The LGAC provides the second level of supervision. The LGAC supervises the VWSs to ensure strict implementation of their work plan. They sometimes monitor the activities of the VCMs as well. The LGACs also conduct advocacy activities and ensure resolution of any rejections encountered by the VCMs. The state officials and CGPP officers also monitor the activities of VCMs and VWSs to ensure adherence to the set targets and to the attainment of polio eradication objectives.

### VCM contributions and reach.

The VCMs conduct social mobilization activities and hold health education sessions at least once a week in their communities. These activities are aimed at improving OPV acceptance, increasing routine immunization coverage, talking with non-compliant parents, and reaching missed children. The CGPP has reached 1.4 million people with social mobilization and health messages since the inception of the project in Nigeria in 2013 (Table 4). This is approximately one-third (32%) of the estimated population of people older than 15 years in the five CGPP implementation states. However, the correct percentage may be considerably higher, as CGPP does not work in the entire states of Kaduna, Katsina, or Kano, and only operates in portions of some LGAs. CGPP does not operate in all of Borno or Yobe states either.

**Table 4:** People reached with social mobilization and health messages in the local government areas (LGAs) of five Northern states of Nigeria

State	Number of people reached with social mobilization and health messages in the implementing LGA/state between 2013 and 2017
Kaduna	25,217
Kano	571,377
Katsina	176,187
Yobe	159,314
Borno	483,171
Total	1,415,266

Source: CORE Group Polio Project/Nigeria project data.

### Polio supplemental and routine immunization.

A look at polio supplemental immunization coverage from 2014 to 2018 demonstrates the impact of the activities of the VCMs, VWSs, and LGACs on Nigeria’s PEI. Intensive mobilization, resolution of non-compliance, and improved engagement of key stakeholders led to a sharp decline in the percentage of missed children, dropping from 4.5% in FY2014 to only 0.8% in FY2018 (Table 5). This is a particularly notable achievement, given the security and accessibility situations in many of the CGPP focal areas. Each year, more children in CGPP focal areas were vaccinated than in the previous year, growing from 355,148 (FY2014) to 837,454 (FY2018). The CGPP VCMs supported vaccinators during this period in administering more than 1.7 million doses of OPV to children at the time of supplemental immunization campaigns.

**Table 5:** Supplemental immunization coverage for polio, FY2014–FY2018 in CGPP focal areas

Fiscal year	Average annual coverage of polio supplemental campaigns (%)	Number of children immunized with OPV during supplemental campaigns	Number of children younger than 5 years in the population
FY14	95.5	355,148	371,883
FY15	97.1	400,157	412,108
FY16	98.9	452,197	457,226
FY17	98.5	494,359	501,887
FY18	99.2	837,454	844,208

Source: CGPP Final Evaluation Report 2017^21^; CGPP/Nigeria project data.

The population of children younger than 5 years in CGPP focal areas also grew steadily over the period from FY2014 to FY2018 because of births, migrations, and expansion of the program area. In FY2018, there was a sharp increase in the number of children vaccinated. During February and March 2018, a new micro-census was carried out across all settlements. Where possible, adjoining settlements were added to the CGPP programming area, increasing the number of households reached by VCMs. In addition, the program expanded to new LGAs in Katsina and Yobe, resulting in a large increase in the number of children in CGPP focal areas. In addition, there were more SIA rounds. VCMs worked tirelessly to mobilize these expanded populations and to ensure that all children received polio vaccination.

In addition to social mobilization and providing families with information about vaccination, VCMs track the routine immunizations of children within their purview through home visits. More than 85% of caregivers in CGPP focal areas reported that their assigned VCM visited their family on days outside of immunization campaign days.^21^ During home visits, VCMs review immunization cards and discuss polio and other routine immunization with caregivers. When they identify children who are not up to date on routine immunization, they refer them to health centers. During the period from FY2014 to FY2018, CGPP VCMs referred 312,932 children to health centers for routine immunizations (Table 6).

**Table 6:** Number of children younger than 5 years in CGPP areas identified to be in need of routine immunization and referred, FY2014–FY2018

State	Number of children younger than 5 years who missed and were referred for routine immunization in the health facility
Kaduna	6,259
Kano	19,365
Katsina	63,764
Yobe	222,313
Borno	1,231
Total	312,932

Source: CGPP/Nigeria project data.

The contributions of VCMs to routine immunization is evident in the rising coverage in CGPP project areas (Table 7). Significant increases are present in the percentage of children vaccinated with OPV0, OPV3, and being fully vaccinated in CGPP focal areas. In addition, the percentage of “zero dose” (or never-vaccinated children) dropped sharply from 45.1% to 1.4%. Although it is not possible to fully attribute these results to the VCMs alone, it is likely that their work of tracking pregnant women, promoting the OPV birth dose and making it accessible, routinely checking immunization cards and referring unvaccinated children contributed to these gains.

**Table 7:** Routine immunization indicators in CGPP focal areas from 2014 to 2017

Indicator	2014 Baseline	2017 Endline
% Of children aged 12–23 months with OPV0	54.9	98.6
% Of children aged 12–23 months with OPV3	47.2	62.3
% Of children aged 12–23 months who are fully immunized	33.0	57.0
% Of children aged 12–23 months who have never been vaccinated against polio (zero dose)	45.1	1.4

Source: CGPP Final Evaluation Report 2017.^21^

During the KIIs, community and women leaders commended the range of VCM activities, from providing health education and holding discussions during compound meetings to participating in naming ceremonies. These important activities underscore the need to provide accurate information to counter inaccurate beliefs on immunization and vaccinations, resulting in a notable reduction in the number of non-compliant households. A community leader from Ungwan Rimi said:

They [the VCMs] are so dedicated to their duty and we know the only way to help them achieve their objectives is to support their activities by, among others, providing town criers to assist in program information dissemination in the communities and following them to visit families who are still non-compliant to persuade them to accept immunization, knowing the accruing benefits to them and the society.

Another community leader from Ungwan Shanu commented:

Before the [CGPP] intervention in the area, there used to be a high mortality rate each year in the area. But due to the awareness from the VCMs, people are now more aware of the importance of antenatal care visits for the pregnant mothers as well as routine immunization for their children. … People are now taking advantage of these services.

During the interview with the women leaders from Ungwan Shanu, they commended the activities of the VCMs such as encouraging women to accept polio and other immunizations for their children, linking them to the health facility, and educating them on other health-related issues. One of the women leaders said:

We have developed absolute trust with the VCMs to the extent that we allow them to take our children to the health center for immunization and for other health benefits.

Another woman leader commented:

They are so hardworking and nice. We call them anytime of the day, especially when we have any health challenge, and they answer us promptly–no matter the time of day. They should be paid very well for the good work they are doing. In fact, they should be promoted, but still be allowed to stay with us.

The women leaders also commended the introduction of health camps during SIAs. The health camps provided other health services and served as a distribution site for drugs such as pain relievers, cough syrup, vitamins, oral rehydration salts (ORS) and zinc (for diarrhea), antimalarials, antibiotics, deworming tablets, and mosquito nets. They also commended the use of incentives such as soaps, detergents, and sweets during SIAs. One woman leader said:

Women look forward to health camps because we receive free services for other health problems, free drugs and free household items.

Further evidence of VCM effectiveness from the primary data collected include the following: resolving chronically non-compliant cases in settlements in the different states and, during compound meetings, the use of dramas and discussions on issues such as routine immunization (including tetanus toxoid immunization); WASH; antenatal care; and nutrition and exclusive breastfeeding. This enabled the VCMs to clear all the misconceptions on immunization, gain the trust of mothers, and motivate them to have their unimmunized children immunized with OPV and other routine vaccines.

The VCMs also delivered a variety of key messages to the households. It has been demonstrated that families in Nigeria pay more attention to polio vaccination when VCMs engage families to discuss health practices such as breastfeeding, malaria prevention, hygiene, and sanitation together with polio.^26^ This may have informed the introduction of the concept of health camps during SIAs. Typically, health camps provide three separate booths: one for OPV, one for the other routine immunizations, and one for general health services, including the provision of ORS, analgesics, and multivitamins. The introduction of health camps during SIAs has encouraged women not only to vaccinate their children against polio and other childhood killer diseases but also to address other health issues.

## DISCUSSION

The CGPP introduced the use of VCMs to mobilize communities to increase vaccine uptake. Each year, about 500,000 children living in high-risk and hard-to-reach locations are reached by the CGPP VCMs in Nigeria. VCMs are specifically engaged to increase awareness, understanding, and acceptance of polio immunization. Thus, the VCMs provide information on the benefits of immunization by correcting false beliefs and rumors, by addressing both valid and invalid concerns that were preventing people from accessing immunization, and by informing people where and when to get immunized.

There has been little to no turnover of VCMs in the project. This has been vital for consistency and allowed for strong lasting connections with the community. VCMs were selected by the community, are members of the communities where they serve, and have established lasting bonds with families. This strong connection and trust have allowed VCMs to succeed in mobilizing families for vaccination and have made them trusted advisors and sources of information. The CGPP endline evaluation demonstrated community reliance of VCMs, with 73% of caregivers in CGPP focal areas reporting that VCMs are the major source of information on polio in their communities.^21^

The VCMs are contributing to the success of polio eradication in Nigeria in many ways: 1) carrying out house-to-house visits between immunization rounds; 2) tracking all pregnant women and their newborns; 3) ensuring that newborns are taken to the nearest health facility for their first immunizations (Bacillus Calmette–Guérin [BCG], hepatitis B vaccine, and polio birth dose [OPV0]) within the first week of birth and for all subsequent vaccinations, and even on occasion accompanying caregivers and their children to routine immunization sessions; 4) generating lists of immunization defaulters for tracking those children who have not received necessary vaccinations; 5) organizing compound meetings to reduce noncompliance; and 6) ensuring that all births and maternal and child deaths occurring in facilities are recorded and shared with authorities.

Polio field staff have acquired remarkable skills that are necessary for the realization of polio eradication goals, and these skills are applicable to public health initiatives beyond polio. According to Goodman et al.,^27^ polio staff provide other system functions such as disease surveillance and support to the health information system. Our findings demonstrate that the VCMs have acquired valuable skills in community engagement, social mobilization, AFP surveillance, and BCC. These competencies are valuable for supporting other public health priorities, including routine immunization, child health, control and elimination of neglected tropical diseases, integrated disease surveillance and response, strengthening the health information system, and control and prevention of non-communicable disease.

The progress in interruption of WVP in Nigeria can, at least in part, be attributed to training and mobilization of VCMs and community members on surveillance, social mobilization, SIAs, data management, and laboratory work. The engagement of Muslim clerics, polio survivors, medical doctors, qur’anic/Islamic schoolteachers, Christian clerics, traditional rulers, and street entertainers have all provided key support in reaching missed children and non-compliant families, in overcoming community resistance to immunization, and reaching people in difficult and security-challenged terrains. Their establishment and involvement have been one of the most important interventions for polio eradication in Nigeria.

The lessons learned and the strategies used by VCMs should serve a resource for future programming to address broader health goals.^28,29^ This article, along with others documenting the contributions of CGPP-supported VCMs in Nigeria,^24,25^ shows that involving respected, trusted local women in health implementation can be highly effective, particularly when they are supported by other community influencers such as traditional rulers, religious leaders, community leaders, women leaders, and other opinion leaders. Providing referrals and information to address pressing needs of families outside of polio helped increase uptake and acceptance of the program and created trust. The VCM strategies were nimble, innovative, and tailored to the communities to address community challenges that arose.

The findings presented in this article have several limitations that should be considered. First, the quantitative data are from project sources as well as from national or subnational administrative data sources. The quality of the data from administrative sources is not controlled by the CGPP and cannot be verified independently. The accuracy of population data in Nigeria remains a challenge because of poor birth registration, inflated census data, insecurity, and inaccessibility. Thus, we are not able to provide precise estimates of population coverage of the CGPP activities. Second, it is important to note that this study emphasizes qualitative data collected from a subset of CGPP project volunteers and may not be representative of the views of all VCMs and supervisors, or of community mobilizers outside the CGPP. Although our data cannot provide comparisons between program and non-program areas, they do serve to give rich contextual information about how VCMs are selected and trained, and how the work has contributed to polio eradication in Nigeria. The results from our study indicate that community mobilization is possible in conflict-affected areas by using CGPP community volunteers. This study cannot establish a quantifiable measure of the impact of community mobilization efforts. Additional quantitative data collection and analysis are needed to do so. However, documenting the history and contributions of VCMs allows the public health community to understand the evolution of community mobilization work in Nigeria and how the innovative strategies used might be adapted to other health initiatives.

## CONCLUSION

As Nigeria continues its efforts to maintain the interruption of WPV and remains without cases of WPV, there is a continued need to strengthen the routine immunization system and improve immunization coverage. Routine immunization coverage remains inadequate in the Northern states and must be addressed to ensure the eradication of polio and guarantee the health of children. The continued use of VCMs in immunization activities is paramount to this attainment. VCMs have clearly been instrumental to the success of the PEI in Nigeria, and they have supported and improved routine immunization coverage. They have built trust within communities and have provided strong linkages between communities and the primary health-care system. As part of the polio legacy, they are uniquely positioned to support routine immunization and to provide health education, data collection, active disease surveillance, and home visits for hard-to-reach populations for other health initiatives once polio is no longer endemic in Nigeria.

A significant human infrastructure has been built by the PEI in Nigeria: an expansive surveillance network, the use of micro-plans and global positioning system technologies to identify communities in hard-to-reach areas, and enhanced routine immunization management capacity at state, local, and ward levels. These components are available to be transitioned to other health initiatives once polio is eradicated. Documenting these achievements is essential. The documentation, including publication and adoption of polio best practices, will contribute not only to the legacy of polio eradication in Nigeria and beyond but also to faster attainment of targets and objectives for other priority health programs. This article, and the other journal articles in this series help to meet this need.

## Supplemental appendix

Supplemental materials

## Footnotes

† Permanent vaccination teams are also known as “Special Teams” that are mostly used to reach children in mobile populations (e.g., nomads) with immunization services (mostly OPV). They are usually stationed in strategic locations through which these mobile populations transit, ensuring children are vaccinated.
