# Supplementary material for: Contributions of Volunteer Community Mobilizers to Polio Eradication in Nigeria: The Experiences of Non-governmental and Civil Society Organizations
Source: Am J Trop Med Hyg. 2019 Oct;101(4 Suppl):74–84. doi: 10.4269/ajtmh.19-0068 (PMC6776094; doi:10.4269/ajtmh.19-0068)
Supplement: Supplementary file 1 [file tpmd190068.SD1.pdf]

Supplemental Appendix: Questionnaires Used

Research Questionnaire for VCM Supervisors

Letter of Consent

Dear Sir/Madam,

I am a researcher working for Core Group Polio Project in Nigeria to assess the role and relevance of VCMs working with NGOs and CSOs, their performance, and their contributions to the Polio Eradication Initiative (PEI) in Nigeria.

I cordially invite you to contribute to our study by responding to the questions below. The questionnaire should take approximately 20 minutes to complete. Please feel at ease in providing your response. The information provided will be treated as confidential and used for this purpose only.

Thank you and best wishes.

Janefrancis Duru

20 Accept to respond ☐   Will not respond ☐

QUESTIONNAIRE ON THE CONTRIBUTIONS OF VOLUNTEER COMMUNITY MOBILIZERS (VCMs) TO POLIO ERADICATION IN NIGERIA

For Ward Focal Persons - Supervisors

Name of State ____________________

A. VCM SELECTION/COMPOSITION/DEPLOYMENT

1. Do you work with the Volunteer Community Mobilizers (VCMs) for the polio project?
Yes No

10. Which methods did you use to garner community support and commitment for the project?
- Advocacy visits
- Social Mobilization
- Community forum
- Project Champions
- Others, pls list __________________________

____

2. Who were selected as Volunteer Community Mobilizers? (Tick as many as applied)
- Unemployed graduates
- Retired teachers
- Retired health workers
- Religious leaders
- Women leaders
- Opinion leaders
- Traditional leaders
- Others (Specify)……………………………………………………………………………………………

3. What criteria were used in selecting them? (Tick as many as applied)
- Level of Education
  - Elementary 6, SSCE holder, OND/NCE holder, Graduate
- Residence in the community
- Respected in the community
- Speaking native language fluently
- Knowledge of Polio immunization
- Others (Specify)……………………………………………………………………………………………

4. How many of the Volunteer Community Mobilizers (VCMs) are females and males?

- Female ____________

- Males ____________

5. Did the VCMs work in teams?

Yes No

If yes how many persons per team?

……………………

6. Were the VCMs trained before they started work as a community mobilizer?

Yes No

If yes how many times were they trained?

……………………

7. How many Communities/Households were assigned to each VCM team?

Communities____________ Households ________________

8. How many hours did the VCMs work in a week?

- 2- 3hours
- 4 -6hours
- _____________

13. Were the VCMs paid any stipend/allowance? (tick)

Yes No

B. SERVICES PROVIDED & METHODS USED BY VCMS

9. What services did the VCMs render to the Communities? Tick as many as applied.

- House-to-house mobilisation for polio and routine immunization
- Conduct community based AFP surveillance
- Promote routine immunization
- Track the vaccination status of under-fives newborns and pregnant women
- Interpersonal counselling on immunization
- Identify and track chronically missed (unimmunized) children and non-compliant parents (parents refusal of oral polio vaccine)
- Data collection on immunization
- Health education on treatment of diarrhoea, prevention of malaria, breastfeeding, etc.
- Referral
- Others____

____
____
____

11. Where did the VCMs deliver their messages?
   - Households
   - Village square
   - Village meetings
   - Churches/Mosques
   - Schools
   - Motor parks
   - Markets
   - Transit sites (such as major intersections, bus depots and train stations)
   - Others, pls list ___________________________

____

12. What methods were used in delivering messages?
   - Radio advertising/ Jingles
   - Awareness rising campaigns with traditional & religious leaders
   - Word of mouth messages
   - House –to- house visits
   - Compound meetings
   - Town criers
   - IEC materials- posters, stickers, leaflets, etc.
   - Short dramas
   - Community meetings
   - Others, pls list ___________________________

____

14. Did the VCMs use behavioural change communication (BCC) tools to work? (tick)

   Yes No

   If yes, list the behavioural change communication tools they used to work in the communities?

   1. ____
   2. ____
   3. ____

4. ____
5. ____

15. What issues were addressed in the behavioural change communication (BCC) tools you used (tick as many as applied)
    - General knowledge of the vaccination program
    - Importance of vaccination
    - Oral Polio Vaccine (OPV) Safety
    - Immunization population
    - The dose to be administered
    - The route of administration
    - Social mobilization issues
    - Others, pls list _________________________

____

____

16. What is the number of people the VCMs reached with social mobilization/health messages in their communities?

____

17. Number of under-five missed children seen and referred for immunization by the VCMs

____

18. What are the reasons for missed children during immunizations?
    - ‘Child absent’
    - non-compliance
    - Religious belief
    - Refusal
    - Others, specify

    ____

    ____

19. Did the VCMs work with the health facilities, traditional birth attendants, traditional healers, traditional leaders, and heads of households in your communities?

    Yes No

    If yes, how many times did they visit them?

State	No of Visits to Health Facilities	No of Visits to Traditional Birth Attendants	No of Visits to Traditional Healers	No of Visits to Traditional Leaders	No of Visits to Households

20. Did you conduct heath education talks in your communities?

    Yes No

    If yes, how many times did you conduct health talks in your communities?

____

____

21. Was the project monitored and evaluated?

    Yes No

    If yes by who?

- Project officers
- Community leaders
- LGA officials
- State officials

C. PROJECT RELEVANCE & CONTRIBUTION

22. What informed the use of Volunteer Community Mobilizers (VCMs) for polio eradication in your community?

____

____

____

____

23. What are the contributions of Volunteer Community Mobilizers towards the eradication of polio in Nigeria?

____

____

____

____

24. Why should the contributions of Volunteer Community Mobilizers to polio eradication be documented?

____

____

____

____

____

25. Any other useful information on the contributions of volunteer community mobilizers in polio eradication in Nigeria?

____

____

____

____

____

THANK YOU.

Research Questionnaire for State Coordinators

Letter of Consent

Dear Sir/Madam,

I am a researcher working for Core Group Polio Project in Nigeria to assess the role and relevance of VCMs working with NGOs and CSOs, their performance, and their contributions to the Polio Eradication Initiative (PEI) in Nigeria.

I cordially invite you to contribute to our study by responding to the questions below. The questionnaire should take approximately 20 minutes to complete. Please feel at ease in providing your response. The information provided will be treated as confidential and used for this purpose only.

Thank you and best wishes.

Janefrancis Duru

Accept to respond ☐ Will not respond ☐

QUESTIONNAIRE ON THE CONTRIBUTIONS OF VOLUNTEER COMMUNITY MOBILIZERS (VCMs) TO POLIO ERADICATION IN NIGERIA

For State Project Coordinators

Name of State ____________________

A. PROJECT LGAS/SUPPORT

1. How many Volunteer Community Mobilizers (VCMs) were selected & trained for the project in your State? _________________________

2. List the LGAs selected in your State for the project and the no of VCMs assigned

S/N	Names of LGA selected in the State	No of VCMs used per LGA

3. What were the criteria used in selecting the LGAs supported by the project?

____

____

____

4. Which methods were used to garner stakeholders support and commitment in your State?
- Advocacy visits
- Social Mobilization
- Community forum
- Project Champions
- Others, pls list __________________________

____

B. VCM SELECTION/COMPOSITION/DEPLOYMENT

5. Who were selected as Volunteer Community Mobilizers (VCMs) in your State for the project? (Tick as many as applied)
   - Unemployed graduates
   - Retired teachers
   - Retired health workers
   - Religious leaders
   - Women leaders
   - Opinion leaders
   - Traditional leaders
   - Others (Specify)………………………………………………………………………………………………

6. What criteria were used in selecting them? (Tick as many as applied)
   - Level of Education
     - Elementary 6, SSCE holder, OND/NCE holder, Graduate
   - Residence in the community
   - Respected in the community
   - Speaking native language fluently
   - Knowledge of Polio immunization
   - Others (Specify)……………………………………………………………………………………………….

7. How many of the Volunteer Community Mobilizers (VCMs) in your State are females and males?
   - Female _____________
   - Males _____________

8. How many trainings were organised for the VCMs in your state since the project inception and how many VCMS were trained?

S/N	Title of the Training	No of Trainings Organised	No of VCMs that Attended
	**Total**		

9. How Households/Communities were assigned to each VCM team?

- Communities____________ Households ________________

10. Did the VCMs work in teams?

Yes No

If yes how many persons per team?

……………………

11. How many hours did the VCMs work in a week?

- 2- 3hours
- 4 -6hours
- _____________

12. Were the Volunteer Community Mobilizers (VCMs) paid stipend/allowance?

Yes No

C. SERVICES PROVIDED & METHODS USED BY VCMS

13. What services were rendered by Volunteer Community Mobilizers (VCMs)? Tick as many as applied.

- House-to-house mobilisation for polio and routine immunization
- Community based AFP surveillance
- Promote routine immunization
- Track the vaccination status of under-fives newborns and pregnant women
- Interpersonal counselling on immunization
- Identify and track chronically missed (unimmunized) children and non-compliant parents (parents refusal of oral polio vaccine)
- Data collection on immunization
- Health education on treatment of diarrhoea, prevention of malaria, breastfeeding, etc.
- Referral
- Others____
  ____
  ____
  ____

14. Where did the VCMs deliver their messages?

- Households
- Village square
- Village meetings
- Churches/Mosques

- Schools
- Motor parks
- Markets
- Transit sites (such as major intersections, bus depots and train stations)
- Others, pls list ___________________________

____

15. What methods were used in delivering messages?
    - Radio advertising/ Jingles
    - Awareness rising campaigns with traditional & religious leaders
    - Word of mouth messages
    - House –to- house visits
    - Compound meetings
    - Town criers/announcers
    - IEC materials- posters, stickers, leaflets, etc.
    - Short dramas
    - Community meetings
    - Convergent messaging
    - Others, pls list ___________________________

____

16. Did the VCMs use Behavioural Change Communication (BCC) tools to work? (tick)

    Yes No

If yes, list the Behavioural Change Communication tools they used to work in the communities?

6. ____
7. ____
8. ____
9. ____
10. ____

17. What issues were addressed in the Behavioural Change Communication (BCC) tools used by the VCMs? (tick as many as applied)
    - General knowledge of the vaccination program
    - Knowledge of key Household practices
    - Importance of vaccination
    - Oral Polio Vaccine (OPV) Safety

- Immunization population
- The dose to be administered
- The route of administration of common vaccines
- Social mobilization issues
- Prevention of common Health problems

- Others, pls list ___________________________

____

____

18. How many people were reached with social mobilization/health messages in the LGAs supported in your state by the VCMs since the project inception?

S/N	Names of LGA selected in the State	No of People reached with social mobilization / health messages

19. Number of under-five missed children seen and referred for immunization since the project inception.

S/N	Names of LGA selected in the State	No of under-five missed children seen and referred for immunization in the LGA

20. What are the reasons for missed children during immunizations?
    - ‘Child absent’
    - non-compliance
    - Religious belief
    - Refusal
    - Others, specify ________________________

    ____

    ____

21. Did the VCMs work with the health facilities, traditional birth attendants, traditional healers, traditional leaders, religious leaders and heads of households in their communities?
    Yes No

    If yes, how many times did they visit them since the project inception?

Name of LGA	No of Visits to Health Facilities	No of Visits to Traditional Birth Attendants	No of Visits to Traditional Healers	No of Visits to Traditional Leaders	No of Visits to Households
**Total**					

22. Did the VCMs conduct heath education talks in their communities?
    Yes No

    If yes, how many times did they conduct health talks in their communities in a week?

____

23. What are the objectives of polio communication interventions in Nigeria? Tick all that applies
    - Advocacy

- Social mobilization
- Behavioural change communication

24. How did you supervise and monitor the activities of VCMs?

____

____

____

____

D. PROJECT RELEVANCE & CONTRIBUTION

25. When did CGPP start working in Nigeria…………………… and for how long now

………………………………………………..?

26. List other CGPP Implementing Partners that worked with you if different from above

____

____

____

27. List the CSOs/NGOs that worked with you in your State.

1. ____
2. ____
3. ____

28. What informed the use of Volunteer Community Mobilizers (VCMs) for polio eradication in your State?

____

____

____

____

29. What are the innovative strategies employed by the Volunteer Community Mobilizers for polio eradication in Nigeria?

____

____

____

____

30. What are the contributions of Volunteer Community Mobilizers towards the eradication of polio in Nigeria?

____

____

____

____

31. Is it necessary to document the contributions of Volunteer Community Mobilizers to polio eradication in Nigeria?

Yes No

If yes give reasons

____

____

____

____

____

32. Any other useful information on the contributions of volunteer community mobilizers in polio eradication in Nigeria?

____

____

____

____

____

THANK YOU.
